# Wide-angle scanning planar array with quasi-hemispherical-pattern elements

**DOI:** 10.1038/s41598-017-03005-3

**Published:** 2017-06-02

**Authors:** Ren Wang, Bing-Zhong Wang, Changhai Hu, Xiao Ding

**Affiliations:** Institute of Applied Physics, University of Electronic Science and Technology of China, Chengdu, 610054 China

## Abstract

A compact quasi-hemispherical-pattern antenna, two linear wide-angle scanning arrays, and a planar wide-angle scanning array are proposed. To increase the field-of-view scanning range of arrays, a compact low-profile antenna with a quasi-hemispherical pattern is introduced firstly. When the ground is infinite, the proposed antenna has a quasi-hemispherical pattern, i.e., an approximately uniform radiation in the upper half space. Afterward, two linear arrays arranged along the length and width of the proposed antenna’s radiation patch are presented. The main beams of the two arrays with 16 active elements can scan from less than −75° to more than +75°. When the linear array element number is 128, the maximum scanning angle can reach 86°. At last, a planar array with 16 × 16 active elements is proposed. In two special planes, *xz* plane and *yz* plane, the main beams of the planar array can scan from less than −77° to more than +77° with a gain fluctuation less than 5 dB and a maximum side lobe level (SLL) less than −10 dB. An excellent wide-angle-scanning performance both in linear and planar arrays can be obtained using the proposed method.

## Introduction

Wide-angle scanning arrays are highly desirable in many military and civil applications, such as modern wireless communication systems, broad-coverage energy transmission systems, and radar systems. However, scanning to low elevation is difficult for both Huygens gradient metamaterials^1, 2^ and planar phased array because the elements’ beam-widths are limited and mutual coupling between elements will be strong when the array scans its beam close to the end-fire direction. For the wide-angle scanning planar phased array, designing broad-beam elements, ideally with a hemispherical pattern, dealing with the strong coupling, and setting small element distance to avoid grating lobes are three main challenges.

To broaden the scanning range of planar arrays, some kinds of methods have been presented. The first one is using reconfigurable antenna elements^3–7^. In the reconfigurable method, the beam-width of the element is not wide, and a large covering range is obtained by changing beam directions of the elements. In ref. 3, a multi-panel approach for phased arrays was introduced to obtain the wide-angle coverage performance by mechanical adjusting. In refs 4–7, some novel pattern reconfigurable elements were proposed to jointly cover a large range. Arrays with reconfigurable elements can scan their main beams up to a maximum of about ±70° in one dimension by dividing the scanning space into multiple regions. Two-dimensional wide-angle scanning arrays are difficult to be realized using reconfigurable method because of the large size and complexity of elements.

Different from the reconfigurable elements method, metamaterial lens method can broaden the scanning range of traditional arrays by covering a metamaterial layer^8–11^. Metamaterials can provide electromagnetic waves with some novel propagation properties by controlling permittivity and permeability independently^12, 13^. A buckyball lens with metamaterials was used to bend the electromagnetic wave and broaden the scanning range of planar arrays in ref. 8 and some simplified metamaterial lenses were proposed in refs 10 and 11. Compared to the reconfigurable elements method, the metamaterial lens method needs no mechanical and electrical adjusting system to switch elements’ beams. However, metamaterial lens is very complex and large, which is undesired for carrier-based and on-board applications.

The third method to design wide-angle scanning arrays mainly focuses on the coupling. In refs 14 and 15, the crossed L-bar and comb-slot microstrip antennas with plate-through holes were proposed to broaden scanning range. The plate-through holes and field-matching rings were arranged to decrease the coupling between elements. Wide-angle scanning up to a maximum of 60° in two dimensions was achieved in the operating frequency bands. In addition, decoupling networks are developed to reduce the mutual coupling between adjacent elements by analyzing scattering matrix in refs 16 and 17. By these decoupling methods, the scanning range can be broadened to approximately 70° without scanning blindness. At present, the decoupling network is just used in one dimensional scanning arrays because the two-dimensional decoupling network is quite complex. In contrast with the decoupling method, tightly-coupling method focuses on using coupling to broaden the scanning range, instead of decreasing coupling^18–20^. The tightly-coupling array should have a complex multi-layer structure and a high profile, because matching networks are necessary for this kind of arrays. Another method regarding coupling is wide-angle-impedance-matching (WAIM) layers, which can match the active impedance of elements at different scanning angles by covering array surface with a thin planar layers^21–23^. The relations between the WAIM variables and the array reflection coefficient are complex and an effective synthesis method is required^21^.

As we have described, broad-beam elements and strong coupling are main challenges for a wide-angle scanning planar phased array. Different from the third method, the fourth method mainly focuses on the other challenge, i.e., wide-beam elements. Ref. 24 pointed out that the boundary condition of ground is the key to design wide-beam elements. By changing the metal ground to artificial magnetic conductor, some one-dimensional wide-angle scanning arrays are proposed in refs 24 and 25. However, antennas in refs 24 and 25 are multi-layer and complex, which is difficult to fabricate and assemble. In addition, these antenna elements are not suitable to be used in two-dimensional wide-angle scanning arrays.

To design low-profile and easy-fabricated planar wide-angle scanning arrays and further increase the scanning range, in this paper, a compact three-magnetic-current (TMC) antenna is proposed firstly. The proposed TMC antenna has a quasi-hemispherical pattern, i.e., an approximately uniform radiation in the upper half space. Afterwards, two linear arrays with different element directions are proposed. The main beam directions of the two linear arrays have a scanning range more than 150° with a gain fluctuation less than 3 dB and a maximum side lobe level (SLL) less than −10 dB. Then, a planar phased array with 16 × 16 active elements is introduced. This array can scan up to 77° and 78° in *xz* plane and *yz* plane, respectively. Excellent wide-angle scanning performances both in linear and planar arrays can be obtained by the proposed method. In addition, the wide-angle scanning arrays with TMC elements have a single-layer substrate and a low profile, which is suitable for carrier-based and on-board applications.

## Design Procedures and Results

### Design procedures

**Figure 1:**
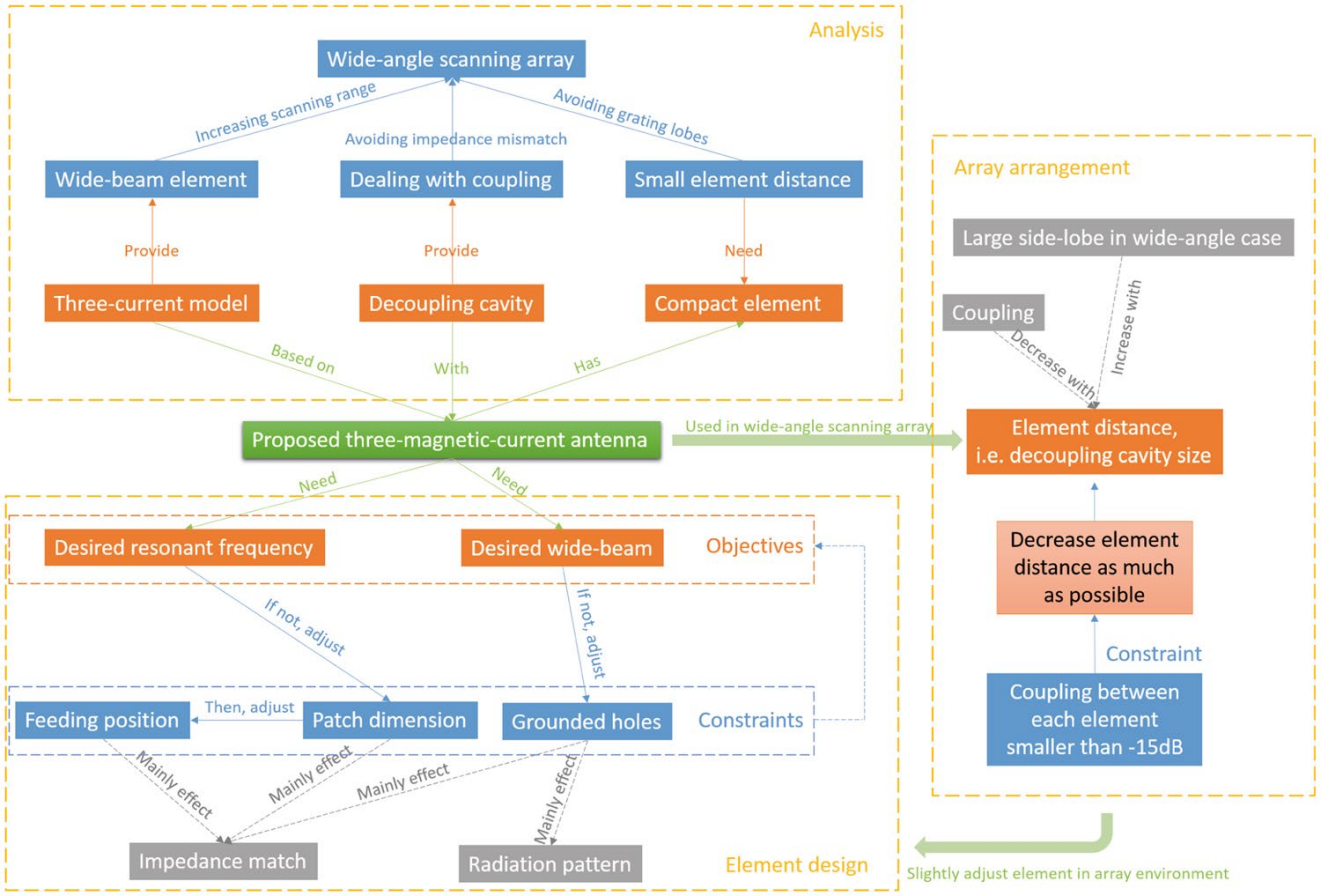
Design methodology of the proposed antenna.

Generally, there are three challenges to design wide-angle arrays: designing wide-beam element, dealing with coupling between elements, and setting small element distance to avoid grating lobes, as shown in Fig. 1. According to the product principle of array pattern, array element with a wide beam is conducive to obtain wide-angle scanning performance. To guide the design of broad-beam antennas, we have proposed a three-current model with a quasi-hemispherical pattern^26^. This model contains two circumstances, the first one is three magnetic currents on an infinite electric wall and the second one is three electric currents on an infinite magnetic wall. Although the structure in ref. 26 can obtain a broad beam, it is not suitable for array applications because of its electrically large dimension. Therefore, we propose a novel compact TMC antenna structure in the paper. To satisfy the requirement of small element distance, typically smaller than half a wavelength, array element should be compact. However, when the element distance is small, the coupling will be strong, especially when the beam scans to near-end-fire directions. Therefore, dealing with coupling is necessary for wide-angle scanning arrays. Grounded decoupling cavity is an effective method to decrease coupling between array element^14, 15^, and it is used in this paper.

The objective of the proposed TMC antenna is to achieve a hemispherical pattern at desired resonant frequency with a structure dimension smaller than half a wavelength. The constraint variables include patch dimension, feeding position, and grounded holes. These parameters have different effects on antenna performances, which will be analyzed in next section to show the design methodology clearly. After antenna element design, we should arrange the elements as an array. To decrease the coupling between elements, the element distance should be as large as possible, although the decoupling cavity is used. However, according the scanning performance of array factor, the side lobe in the near-end-fire scanning cases will increase with element distance, and grating lobe will appear when element distance is larger than half wavelength. Therefore, we should decrease the element distance under the condition that the coupling is acceptable. After determining the element distance, the element structure parameters, patch dimension, feeding position, and grounded holes on patch may need to be slightly adjusted to make sure the element has desired resonant frequency in array environment.

### Compact three-magnetic-current antenna

**Figure 2:**
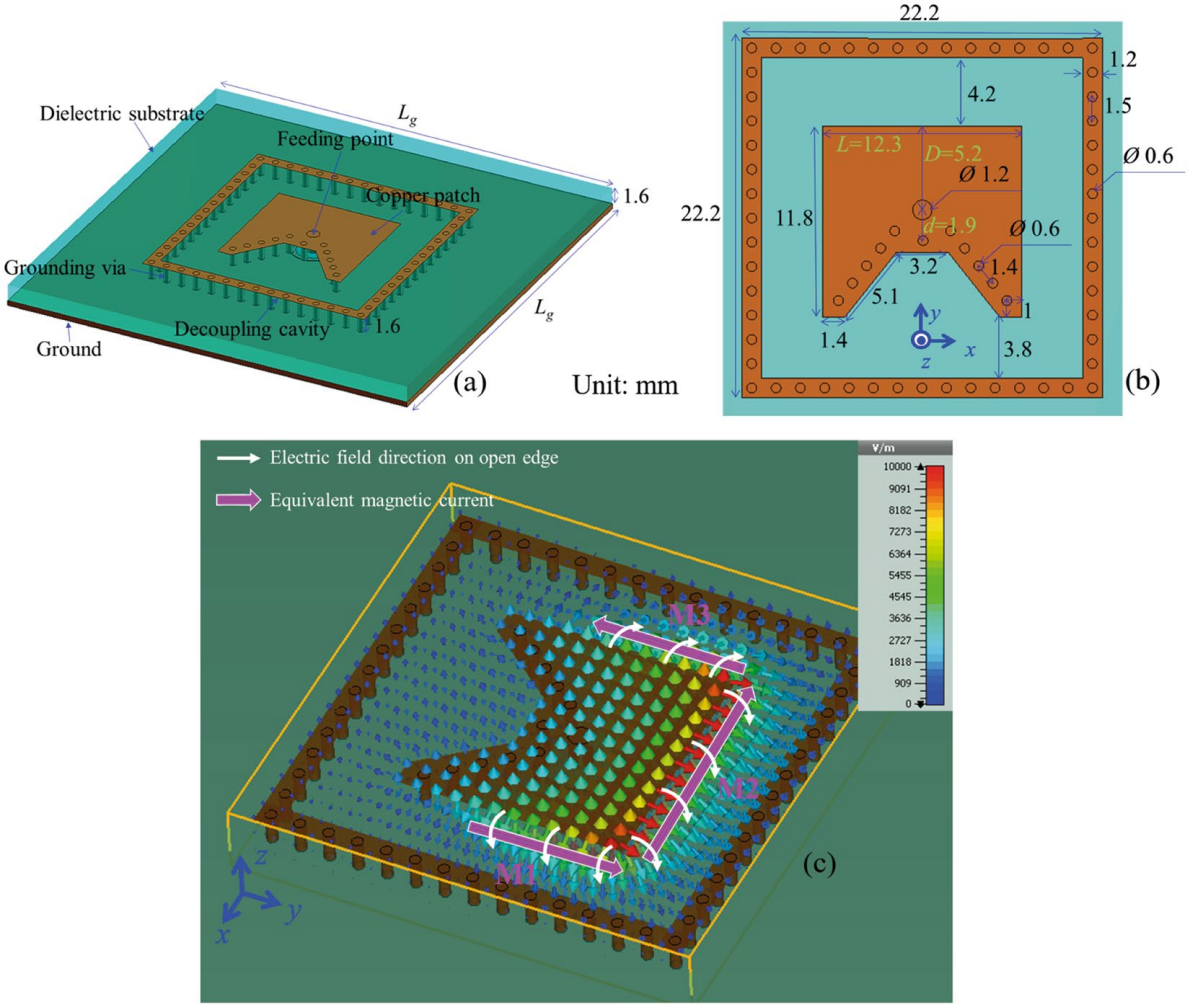
Geometry of the TMC antenna. (**a**) Perspective view and (**b**) partial enlarged detail. The color brown represents copper material and the color aquamarine represents dielectric substrate. (**c**) Electric field distribution. The directed equivalent magnetic currents have been marked in the figure. (Unit: mm).

Geometry of the TMC antenna and its optimum dimensions are shown in Fig. 2. A decoupling cavity composed of a square strip and grounding vias is set around the radiation patch to decrease coupling between elements in arrays. The electric field distribution of the TMC antenna is shown in Fig. 2(c) (also see Movie S1). On the three open edges, electric field is much stronger than the short edge and goes from patch to ground. According to the principle of equivalence, electric field on open edges of slot antennas can be equivalent to magnetic current:

1 $${J}_{m}=-{n}_{z}\times {E}_{t}$$

where, *n*_*z*_ is unit vector in the z direction and *E*_*t*_ is the electric field in slots. By analogy with the radiation principle of slot antennas, fields on the three open edges of the patch can be equivalent to magnetic currents^26, 27^, which are marked in the figure. Magnetic current 1 (M1) has an equal amplitude and inverse direction with magnetic current 3 (M3), which compose a binary array. Based on the radiation of short currents and imaging theory, when the ground is infinite, the pattern nulls of the binary array (M1 and M3) should locate at the yz plane, and the pattern maximums should direct to +x and −x. The pattern nulls of magnetic current 2 (M2) should direct to +x and −x, and the pattern maximums of M2 should locate at the yz plane. In other word, the pattern nulls of M2 and the pattern maximums of the binary array have the same directions, and the pattern maximums of M2 and the pattern nulls of the binary array have the same directions. By adjusting the dimensions to equate the M2’s pattern maximum with the binary array’s, a two-dimensional broad beam may be obtained.

To show the operation principle further, some main parameters of the proposed antenna are analysed in the following. In all the following discussions, the ground is set as infinite to clearly show the parameter’s effect on radiation patterns and avoid the influence of ground edge.

**Figure 3:**
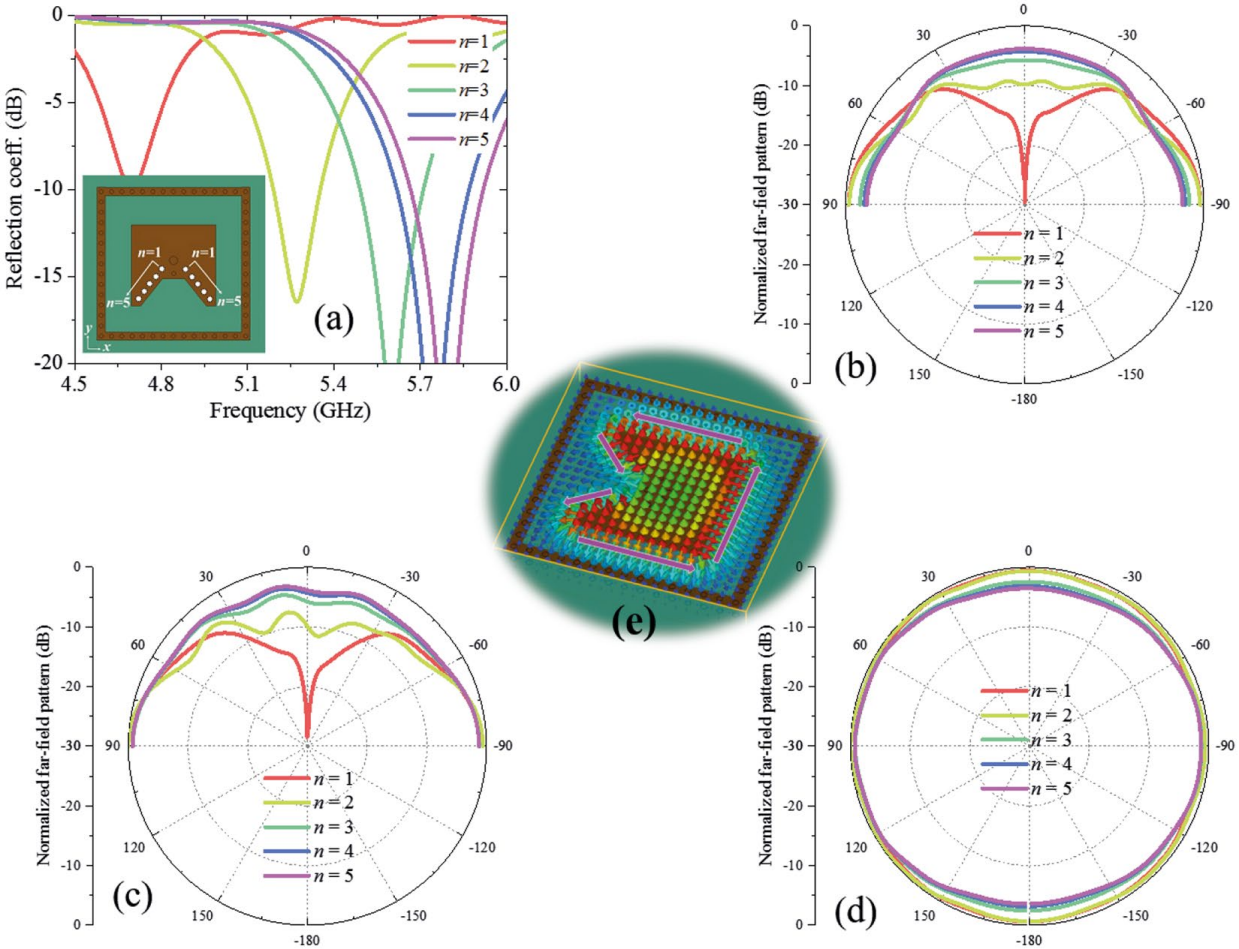
Relations between the number *n* of the grounded holes on short edge and antenna performances. (**a**) Reflection coefficient versus number *n*. The insert figure shows the grounded holes discussed here, which are marked as white circles. The distance between adjacent grounded holes are fixed and positions of the grounded holes are shown in Fig. 2. Normalized radiation patterns in xz plane, yz plane, and xy plane at 5.8 GHz versus number *n* are shown in (**b**–**d**), respectively. With the decreasing of grounded holes, radiation on z-direction decreases. (**e**) Electric field distribution at 5.8 GHz on the case of *n* = 1. The equivalent magnetic currents are marked in the figure, which compose a magnetic current loop. In the calculation, the ground is set as infinite.

From Fig. 2, we can see that the grounded holes are the key to realize three equivalent magnetic currents, therefore, the grounded holes are analysed firstly. Number *n* of the grounded holes on the short edge is discussed in Fig. 3. The distances between adjacent grounded holes are fixed and positions of the grounded holes are shown in Fig. 2. With the number *n* increasing, the short range on edges increases. For example, when *n* = 1, only three grounded holes around the feeding cable exist; when *n* = 5, the tortuous edge of the patch is almost short, which is the case of the proposed antenna in Fig. 2. From Fig. 3(a), the resonant frequency increases with the number *n*. From Fig. 3(b–d), we can see that the radiation along z axis increases with the hole number *n*. When *n* = 1, the radiation along z axis is very small and the pattern looks like the radiation of a monopole along z axis. When *n* = 5, the radiation along z axis increases to the similar level of x and y directions and the pattern becomes quasi-hemispherical. In the case of *n* = 1, the electric field is strong on all the edges of the patch. The equivalent magnetic currents on the edges compose a magnetic current loop, which provides a monopole-like radiation.

**Figure 4:**
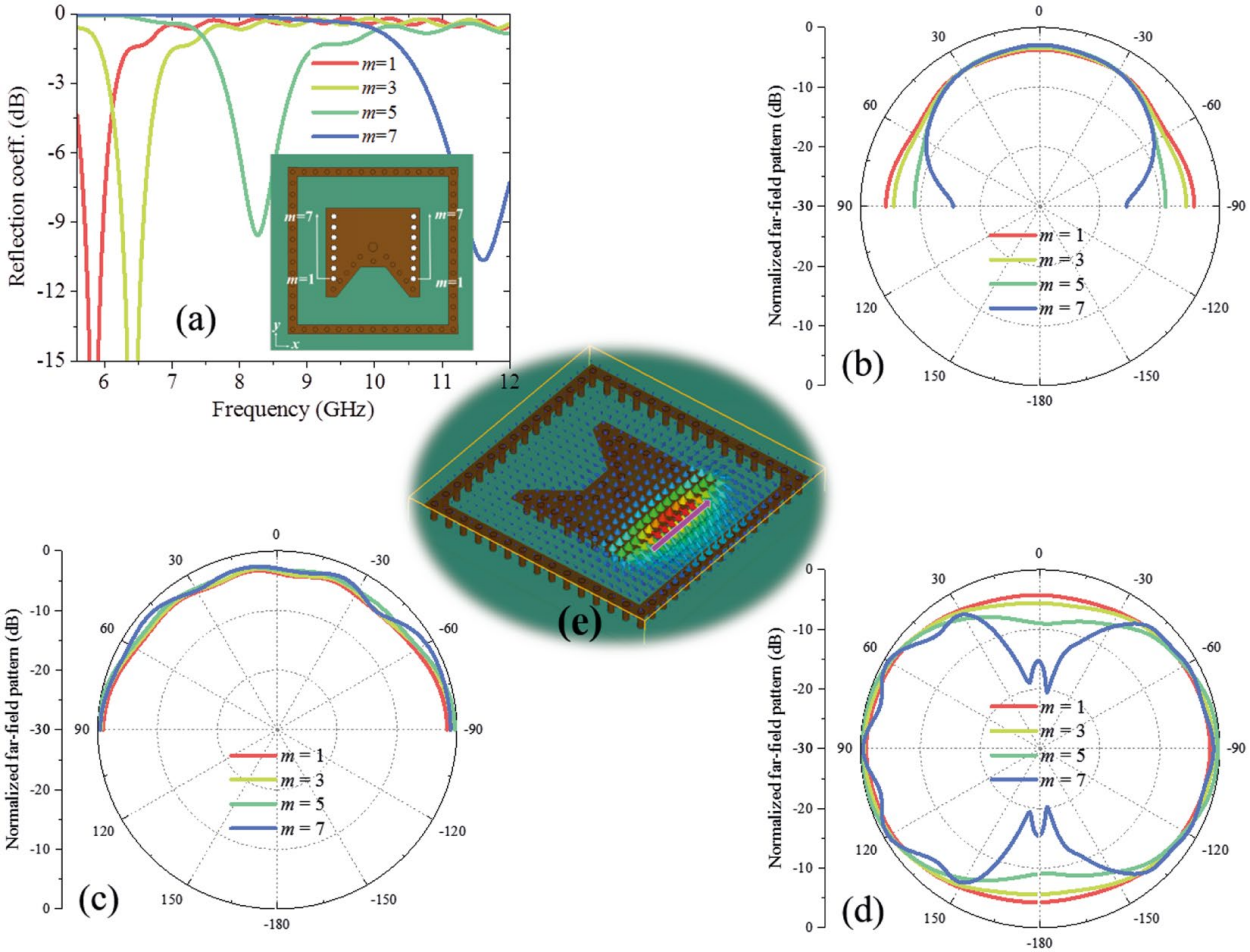
Relations between the number *m* of the additional grounded holes on two side edges and antenna performances. (**a**) Reflection coefficient versus number *m*. The insert figure shows the additional grounded holes discussed here, which are marked as white circles. These grounded holes are not used in the proposed antenna shown in Fig. 2, which is only used to show the operation principle of the structure. The distance of these holes are fixed and the same as the grounded hole on the short edge in Fig. 2. Normalized radiation patterns in xz plane, yz plane, and xy plane at 5.8 GHz versus number *m* are shown in (**b**–**d**), respectively. With the increasing of grounded holes, radiation on x-direction decreases. (**e**) Electric field distribution at 5.8 GHz in the case of *m* = 7. The equivalent magnetic current is marked in the figure. In the calculation, the ground is set as infinite.

Next, some additional grounded holes along two opposite side edges are added to the proposed antenna, as shown in Fig. 4. These additional grounded holes, marked as white circles, are not practically used in the proposed antenna shown in Fig. 2, which is only used to show the operation principle of the structure in the discussion section. The distances of these holes are fixed and the same as the grounded hole on the short edge in Fig. 2. The short range on the opposite side edges increases with the number *m*. When *m* = 1, the opposite side edges are almost open; when *m* = 7, only one edge of the patch is not grounded. From Fig. 4, we can see that the radiation in yz plane changes little with the hole number *m* and the radiation along x axis decreases with the hole number *m*. When *m* = 7, the radiation along x axis is very small and the pattern looks like the radiation of a dipole along x axis on the ground. In this case, only one edge is open and the electric field is strong on this open edge, which can be considered as an equivalent magnetic current and provides a dipole-like radiation.

From the above analysis, we can see that the equivalent magnetic current can be changed by the grounded holes, therefore, monopole-like radiation, quasi-hemispherical radiation, and dipole-like radiation, can be realized by magnetic current loop, three magnetic currents, and one magnetic current, respectively. In other word, the operation mechanism of the proposed quasi-hemispherical-pattern antenna is based on three magnetic currents achieved by patch antenna with appropriate grounded holes.

**Figure 5:**
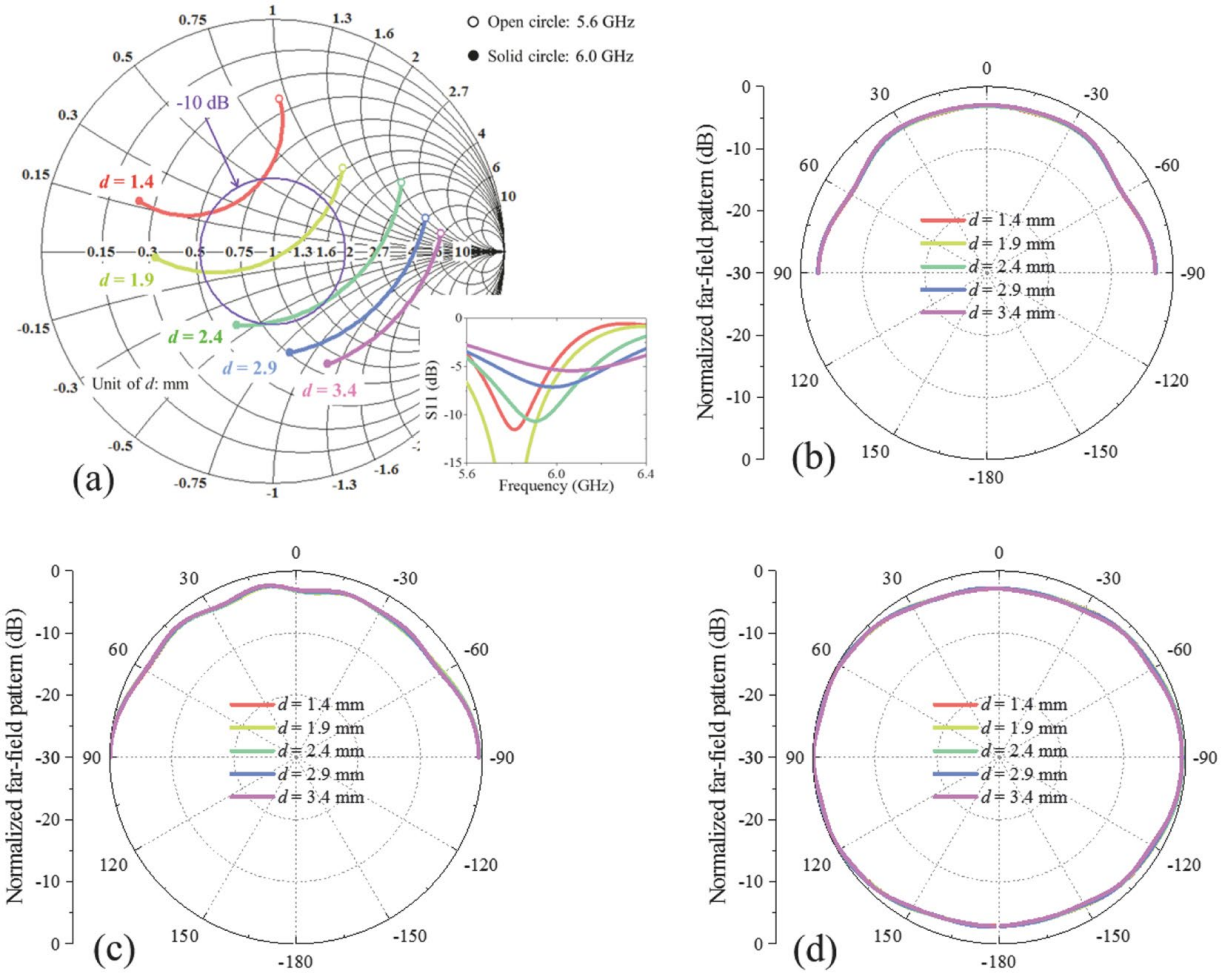
Relations between structure parameter *d* and antenna performances. (**a**) Impedance matching versus *d*. The insert figure shows reflection coefficients corresponding to different *d*. Normalized radiation patterns in xz plane, yz plane, and xy plane at 5.8 GHz versus *d* are shown in (**b**–**d**), respectively. Impedance changes a lot and radiation patterns change little with structure parameter *d*.

**Figure 6:**
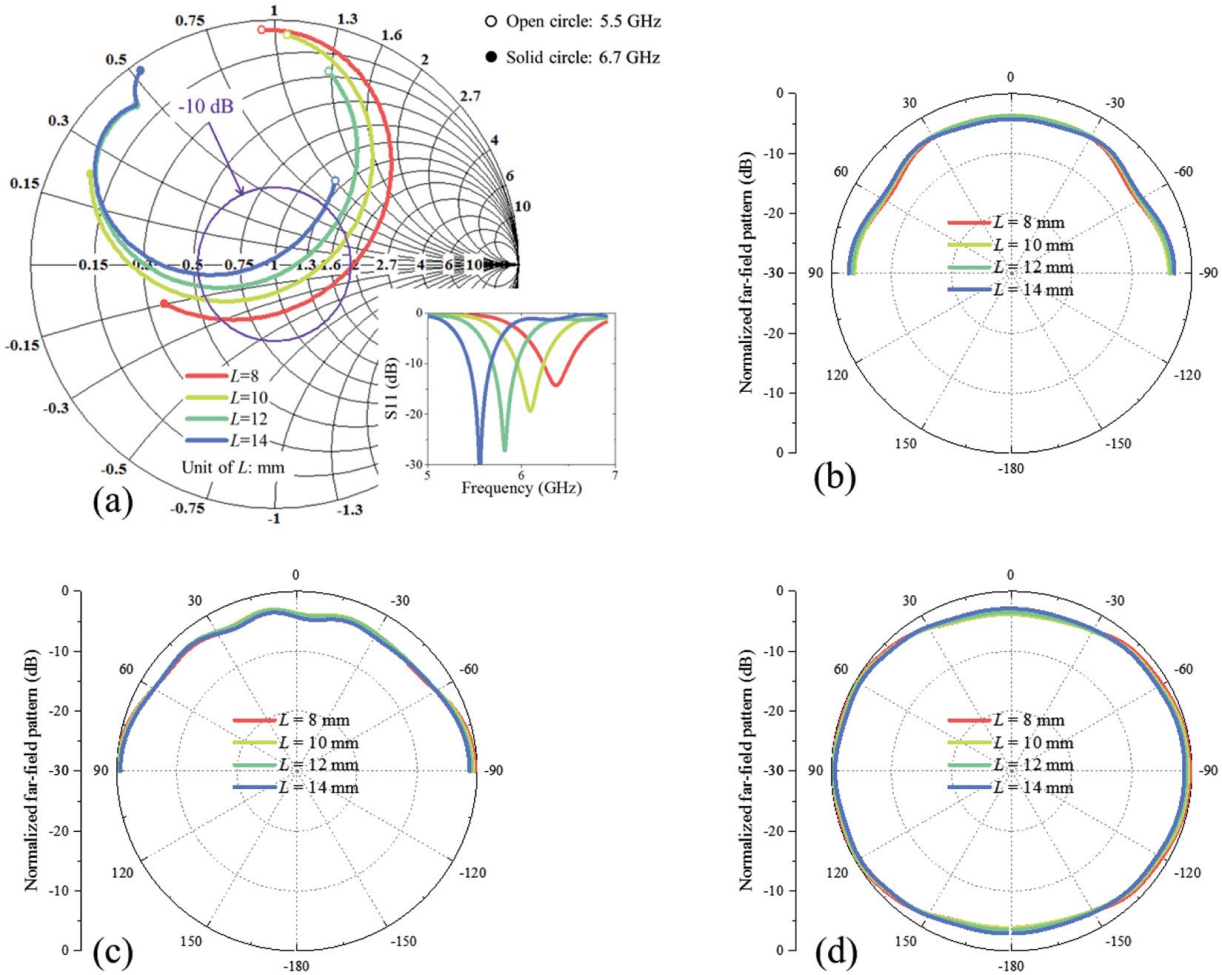
Relations between structure parameter *L* and antenna performances. (**a**) Impedance matching versus *L*. The insert figure shows reflection coefficients corresponding to different *L*. Normalized radiation patterns in xz plane, yz plane, and xy plane at 5.8 GHz versus *L* are shown in (**b**–**d**), respectively. Resonant frequency decreases with *L* and radiation patterns change little with *L*.

**Figure 7:**
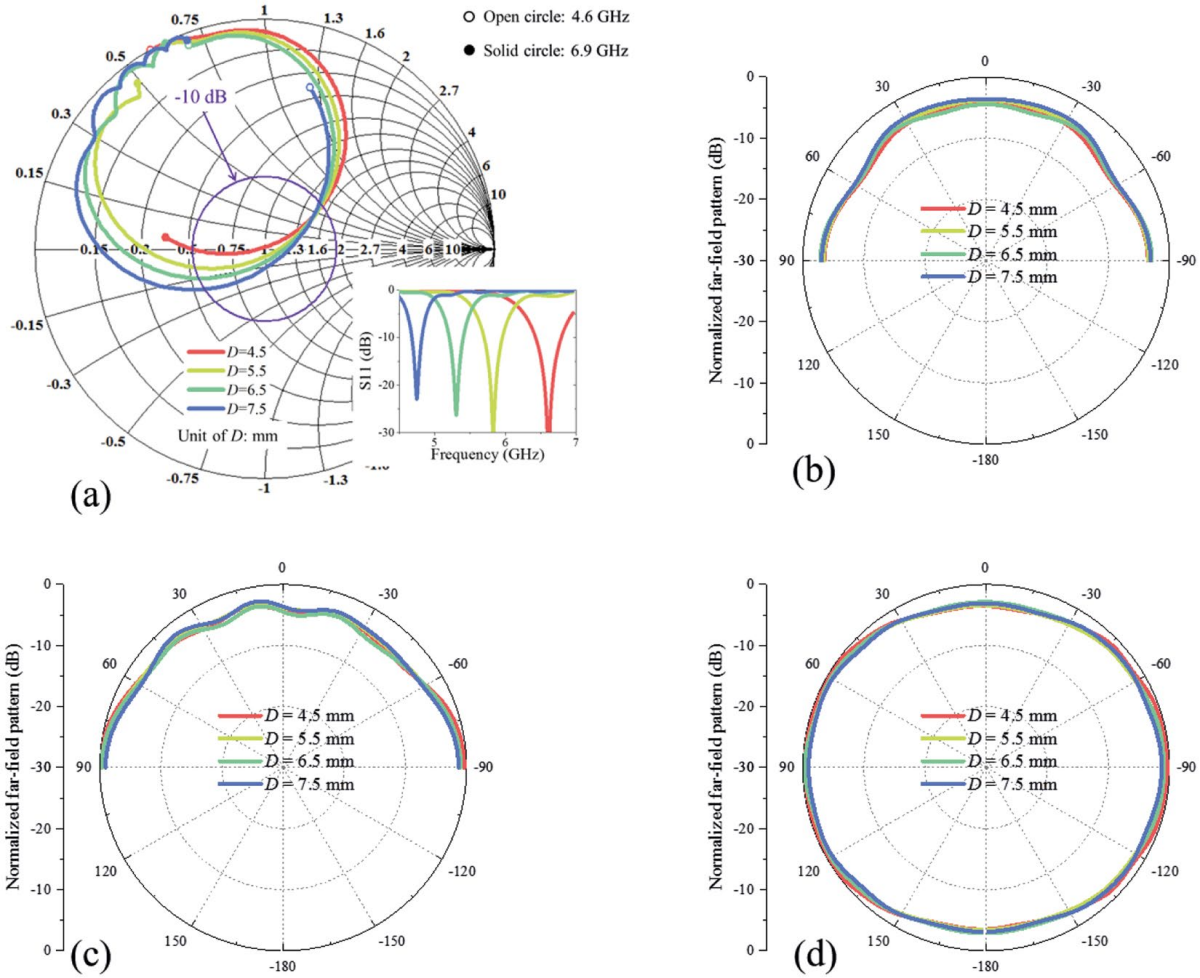
Relations between structure parameter *D* and antenna performances. (**a**) Impedance matching versus *D*. The insert figure shows reflection coefficients corresponding to different *D*. Normalized radiation patterns in xz plane, yz plane, and xy plane at 5.8 GHz versus *D* are shown in (**b**–**d**), respectively. Resonant frequency decreases with *D* and radiation patterns change little with *D*.

Some other key parameters related to structure dimension are analysed next. The first one is *d*, which corresponds to the feeding position. Another two parameters, *L* and *D*, correspond to the size of patch. Figures 5, 6 and 7 show the effects of *d*, *L*, and *D* on antenna performances, respectively. From these figures, we can see that radiation patterns change little with parameter *d*, *L*, and *D*. These three parameters are mainly related to feeding impedance matching: *d* has strong effect on resonant depth and resonant frequency decreases with *L* and *D*.

From above parameter discussions, we can see that different parameters have different effects on the antenna performances. Because structure dimension parameters *d*, *L*, and *D* mainly affect impedance matching instead of radiation patterns, and grounded holes have strong effect on both impedance matching and radiation pattern, this kind of antenna can be designed basically as follows: (1) modelling patch antenna with grounded holes based on three magnetic currents; (2) adjusting dimension parameters to achieve desired resonant frequency; (3) adjusting grounded holes to achieve quasi-hemispherical patterns at desired frequency; (4) resonant frequency may be shifted compared to the result of Step (2) after Step (3), and then repeat Steps (2) and (3), if necessary.

**Figure 8:**
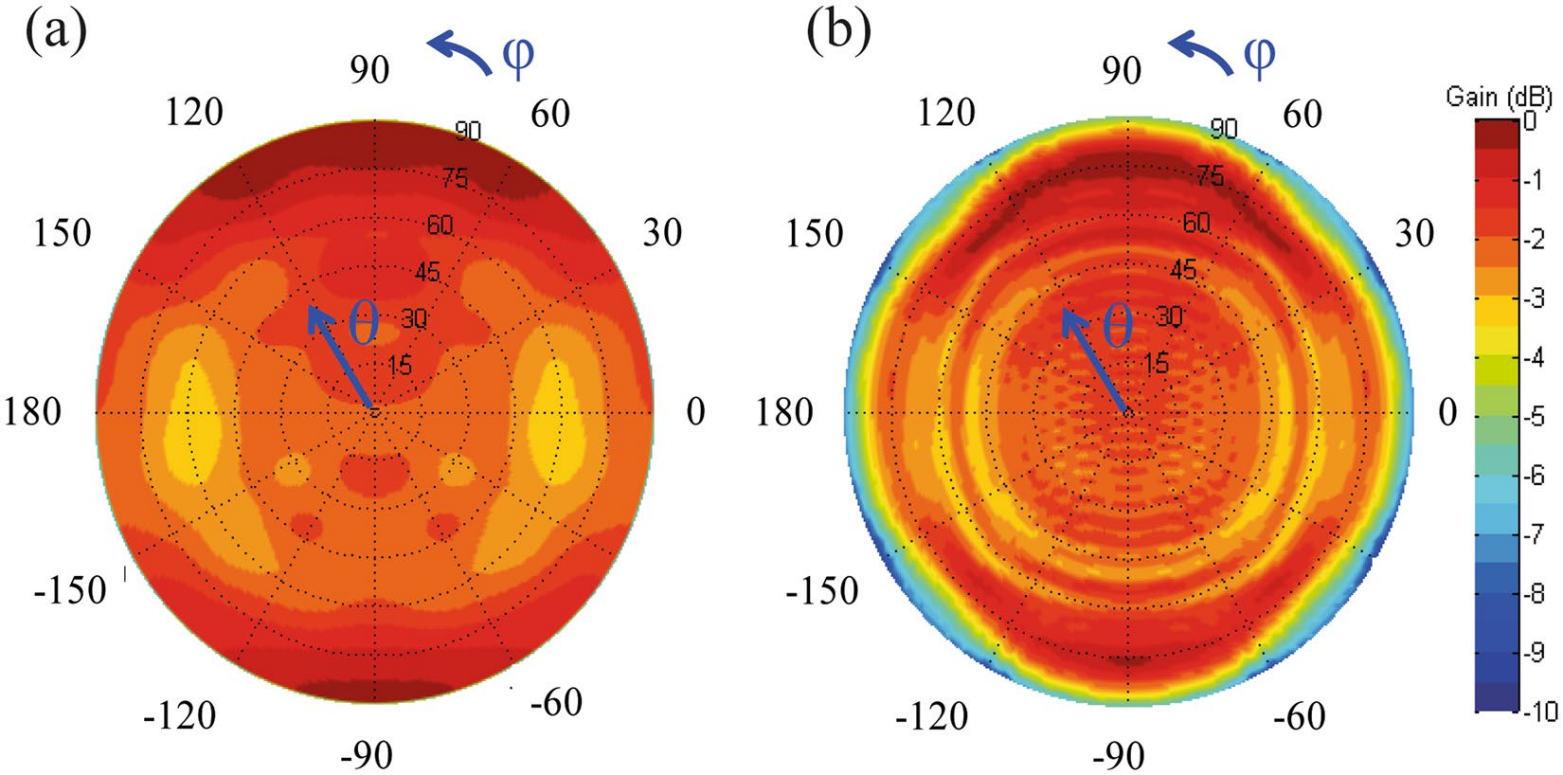
Simulated normalized patterns at 5.8 GHz of the proposed TMC antenna: (**a**) with an infinite ground and (**b**) with a finite ground. The finite ground is a 1200 × 1200 mm^2^ rectangular copper plate.

To display the effect of ground on the radiation, far-field normalized radiation patterns of the proposed antenna with an infinite ground and with a finite ground are shown in Fig. 8(a,b), respectively. When the ground is infinite, the radiation intensity has a fluctuation within 4 dB in the upper half space, which is an approximately uniform distribution. In contrast, when the ground is finite, the radiation intensity in the low-elevation directions decreases to approximately −10 dB and the directions within θ = 80° still have an intensity fluctuation within 4 dB, which represents a two-dimensional wide beam.

**Figure 9:**
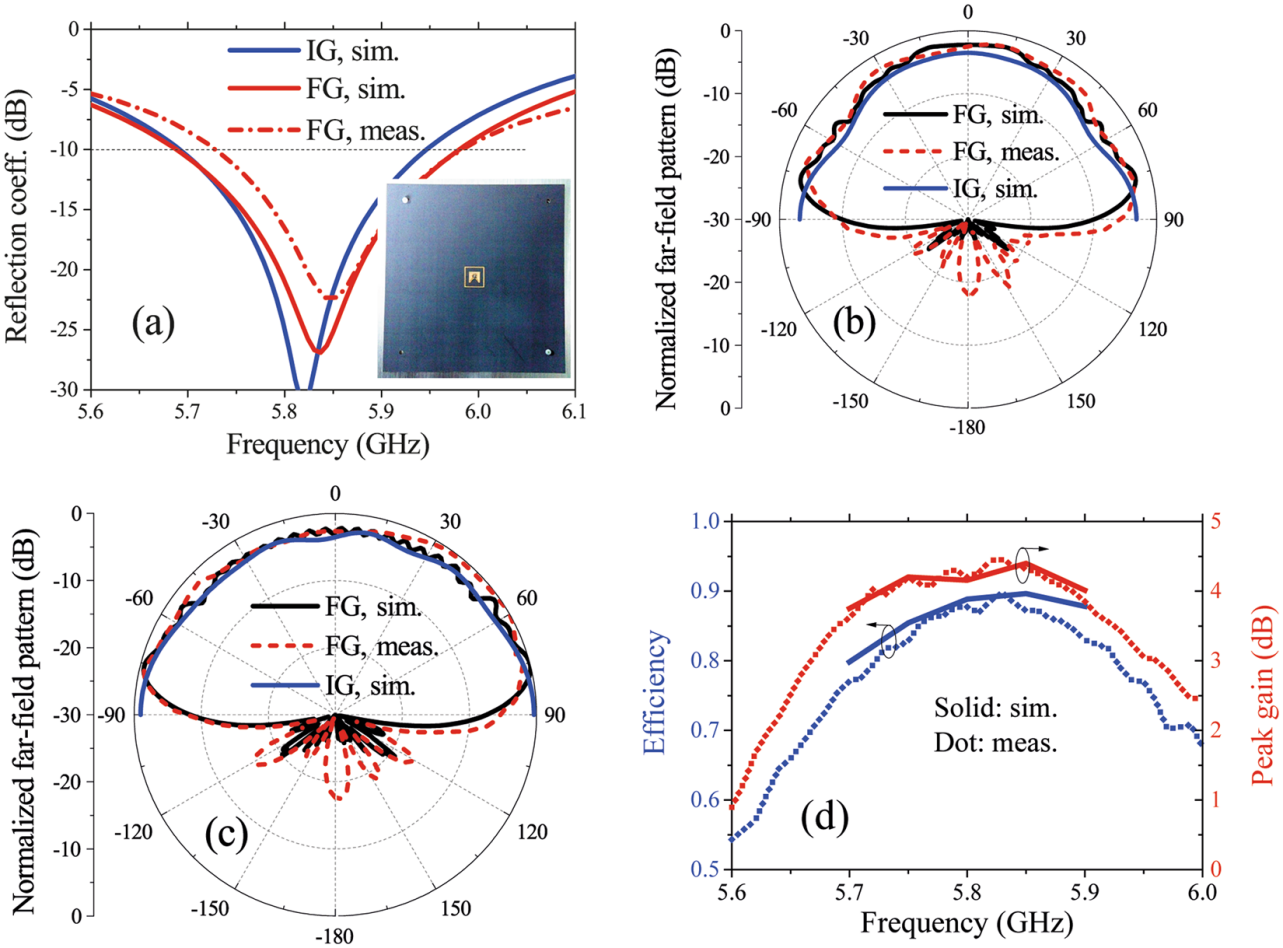
Performances of proposed TMC antenna. (**a**) Simulated and measured reflection coefficients. The embedded figure is a photograph of the TMC antenna, which is fixed on a 1200 × 1200 mm^2^ metal ground with two plastic screws. (**b**,**c**) Far-field patterns at 5.8 GHz in xz plane (**b**) and yz plane (**c**). (**d**) Simulated and measured efficiencies and peak gains when Lg = 1200 mm. IG, FG, sim., and meas. represent infinite ground, finite ground, simulated result, and measured result, respectively.

A prototype of the proposed antenna with optimum dimensions is fabricated and the simulated and measured reflection coefficients are shown in Fig. 9(a). Compared to the infinite ground case, the finite ground has little effects on the reflection coefficient. When the ground is a 1200 × 1200 mm^2^ rectangular copper plate, the simulated band with S_11_ below −10 dB is between 5.69 GHz and 5.97 GHz and the measured one is between 5.73 GHz and 5.97 GHz. The measured resonant frequency has a little difference with the simulated one because of the machining error.

The normalized patterns of the proposed antenna are shown in Fig. 9(b,c). When the ground is infinite, the simulated 3-dB beam-widths in the *xz* plane and *yz* plane are both 180°. When the ground side length *L*_*g*_ is 1200 mm, the simulated 3-dB beam-width in the *xz* plane is 166.5° and the measured one is 170°; the simulated 3-dB beam-width in the *yz* plane is 165° and the measured one is 169°. From the simulated and measured results, we can see that wide beam can be obtained using the proposed TMC structure. In addition, the beam-width increases with the ground size, which is suitable for the wide-angle scanning of large planar arrays. The simulated and measured efficiencies and peak gains of the proposed TMC antenna are shown in Fig. 9(d). The simulated and measured efficiencies are both more than 75% in the band of 5.7 GHz–5.9 GHz.

From the above analysis, we can see that the significant challenge of wide-angle scanning, designing broad-beam elements, can be solved with the proposed TMC antenna. In the next, some wide-angle scanning arrays will be proposed with the TMC elements.

### Linear wide-angle scanning arrays

After antenna element design, we should arrange the elements as an array. The elements are arranged side by side and the decoupling cavities are connected to each other, therefore, the element distance is equal to the side length of decoupling cavity. To decrease the coupling between elements, the element distance should be as large as possible, although the decoupling cavity is used. However, according the scanning performance of array factor, the side lobe level in the near-end-fire scanning cases will increase with element distance, and grating lobe will appear when element distance is larger than half wavelength. Therefore, a compromise between coupling and grating lobe level should be reached by adjusting element distance. One way to reach the compromise is decreasing the element distance under the condition that the coupling is acceptable. In this design example, the acceptable coupling condition is set as coupling between each element smaller than −15 dB. After determining the element distance, the element structure parameters, patch dimension, feeding position, and grounded holes on patch, are slightly adjusted to make sure the element has desired resonant frequency in array environment.

**Figure 10:**
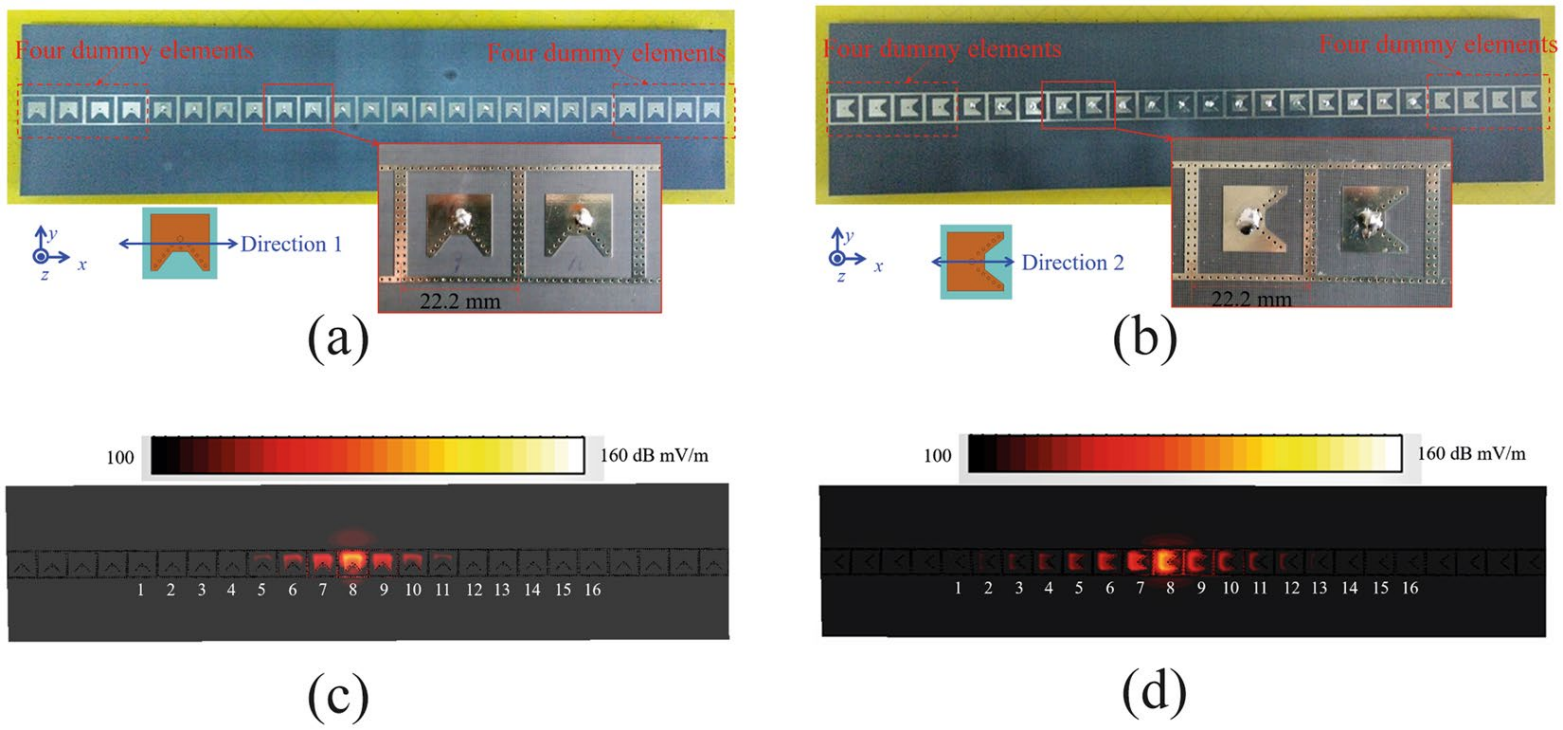
Two linear wide-angle scanning arrays. (**a**) A1 and (**b**) A2. A1 and A2 are respectively arranged along the length (direction 1) and width (direction 2) of the radiation patch. (**c**,**d**) are electric field distribution plots over (**a**) A1 and (**b**) A2 at 5.8 GHz, respectively. The plots are obtained by exciting Element 8 of the arrays, with the other elements terminated in matched loads. Low levels of coupling, below −20 dB, are obtained beyond the second adjacent element from the excited element.

To show the one-dimensional wide-angle scanning performance, two special planar arrays, linear arrays, are introduced in this section, as shown in Fig. 10(a,b). The two linear arrays, Array 1 (A1) and Array 2 (A2), are respectively arranged along the length (direction 1) and width (direction 2) of the radiation patch. The distances between adjacent elements of the two arrays are both 22.2 mm, about 0.43λ, where λ is the free-space wavelength at 5.8 GHz. The two arrays are both composed of 16 active elements and 8 dummy elements. The dummy elements at the ends of arrays can create a similar environment for the active elements to make sure the active elements at different positions with similar active patterns and reflection coefficients. To demonstrate the effectiveness of the decoupling cavity to confine the field distribution within the unit-cell, the electric field distribution plots over the arrays at 5.8 GHz are shown in Fig. 10(c,d). Low levels of coupling, below −20 dB, are obtained beyond the second adjacent element from the excited element, which reveals that the field distributions can be confined well within the unit-cell in the two arrays.

**Figure 11:**
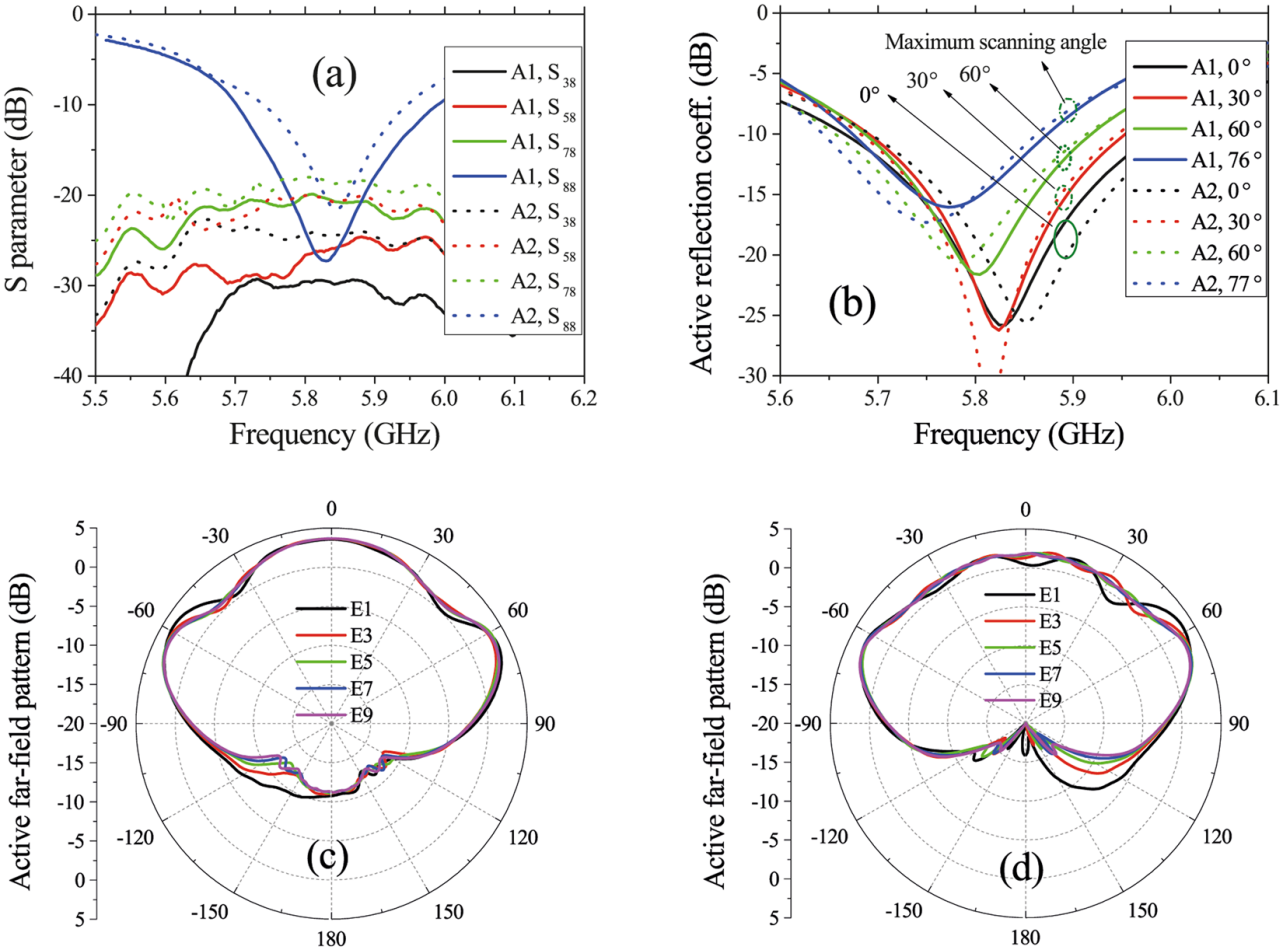
Active S parameters and patterns of the two linear arrays. (**a**) Measured S parameters when Element 8 is excited. (**b**) Active reflection coefficients of Element 8 corresponding to different scanning angles. The active reflection coefficients in the band of 5.73 GHz–5.85 GHz of both the two arrays are below −10 dB in all the scanning status. (**c**,**d**) are measured active patterns in the xz plane of A1 (**c**) and A2 (**d**). E1~E9 represent Element 1~Element 9, respectively.

Some of the measured S parameters of the two arrays are shown in Fig. 11. From this figure, we can see that the insertion losses between any two ports are more than 15 dB, which demonstrates a weak coupling. The active reflection coefficients of the central element (no. 8) are shown in Fig. 11(b). In the figure, the maximum scanning angles for A1 and A2 are respectively 76° and 77°. When the scanning angle increases, the minimums of the active reflection coefficient curves move a little toward low frequency because the coupling will be strong as scanning closed to the end-fire direction. The active reflection coefficients in the band of 5.73 GHz–5.85 GHz of the two arrays are both below −10 dB in all the scanning status. Some measured active patterns in the *xz* plane are shown in Fig. 11(c,d). The 3-dB beam-widths of all the elements in the two arrays are more than 160°. Note that the maximums of the patterns are located at about *θ* = ±70° instead of *θ* = 0°, which will affect the maximum scanning beam directions of the arrays.

**Figure 12:**
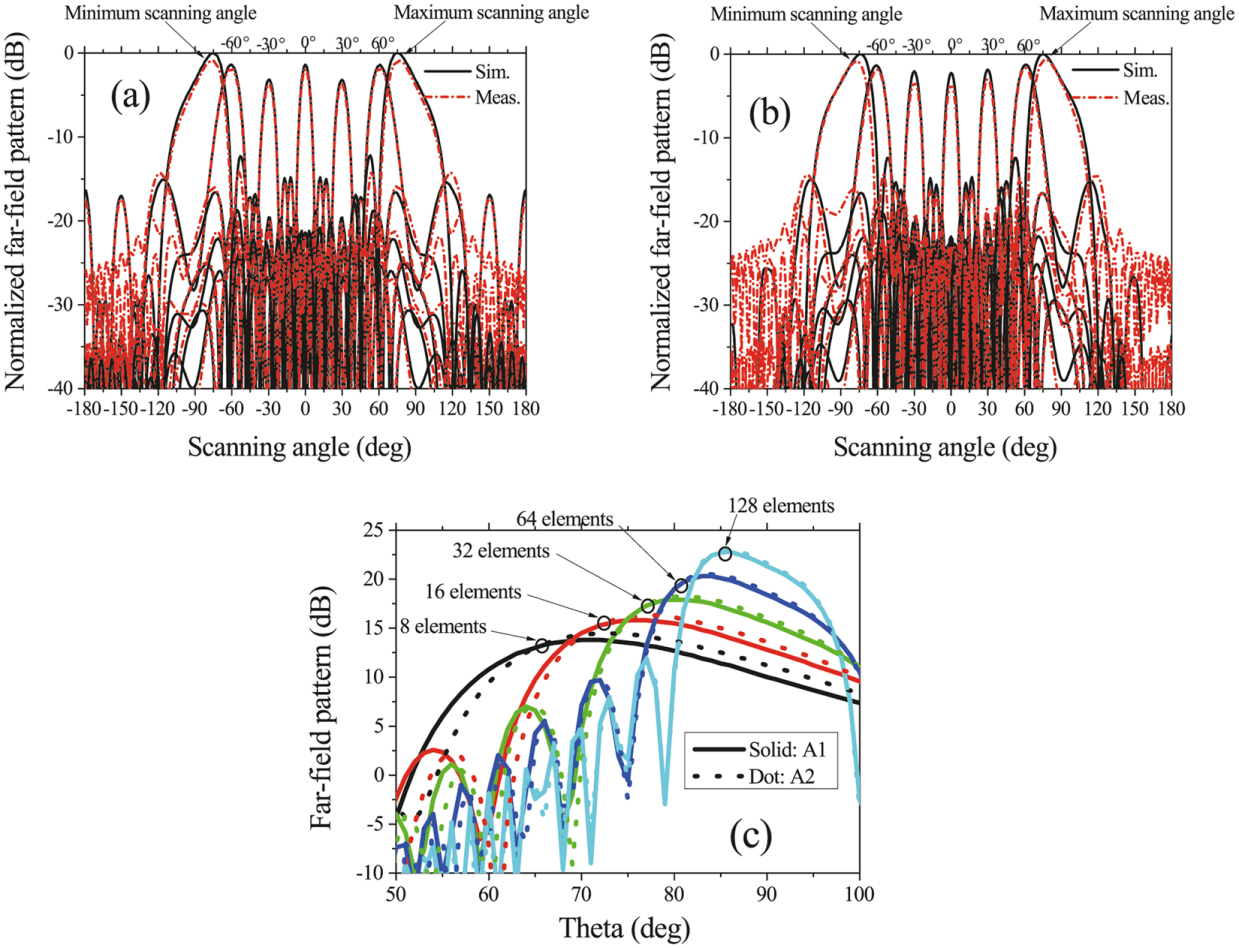
Scanning patterns of the two linear arrays. (**a**,**b**) are scanning patterns at 5.8 GHz in the xz plane of A1 and A2, respectively. (**c**) Simulated patterns in the xz plane at 5.8 GHz of maximum scanning angles when the two linear arrays with different active element numbers.

The simulated and measured scanning patterns of the two arrays in the xz plane at 5.8 GHz are shown in Fig. 12. From Fig. 12, we can see that the two arrays have similar scanning performances. The main beams of A1 can scan from −75° to +75° and scan from −75° to +76° in the simulated and measured results, respectively; The main beams of A2 can scan from −76° to +76° and scan from −77° to +77° in the simulated and measured results, respectively. The simulated and measured scanning gain fluctuations of A1 and A2 are all less than 3 dB; the simulated and measured scanning SLLs of A1 and A2 are all less than −10 dB. The scanning 3-dB beam-widths of the two arrays can both cover the range more than 180°. Because the distance between adjacent elements is smaller than 0.5λ and the maximums of active patterns are located at approximately θ = ±70°, the scanning beams with maximum gains of the arrays do not direct to the broadside direction. Figure 12 also shows that the maximum scanning angles are not ±90°, which is limited by the array element number. Figure 12(c) shows the relation between maximum scanning angle and element number. The maximum scanning angle increases with the element number in both of the two linear arrays. When the array element number is 128, the maximum scanning angle can reach 86°.

From the above discussions, we can see that one-dimensional wide-angle scanning can be obtained with the proposed compact TMC elements. In addition, the two linear arrays along different directions of radiation patch have similar performances. In the planar array, two-dimensional wide-angle scanning is desired and the scanning performances in different scanning planes had better be similar. The different scanning planes are corresponding to different directions of radiation patches, which can be revealed by the two linear arrays’ similar performances. A planar array with 16 × 16 active elements is proposed in the next section.

### Planar wide-angle scanning array

**Figure 13:**
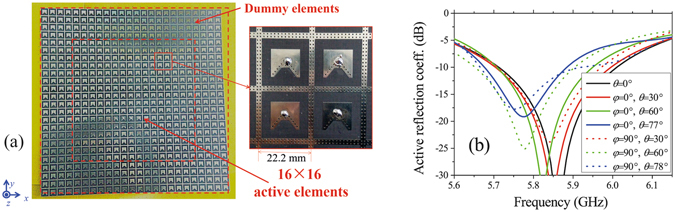
Planar wide-angle scanning array with TMC elements. (**a**) Geometry of the planar array. (**b**) Measured active reflection coefficients of Element (8, 8) corresponding to different scanning angles. The active reflection coefficients in the band of 5.73 GHz–5.86 GHz are below −10 dB in all the scanning status.

A planar wide-angle scanning array with the proposed TMC elements is shown in Fig. 13(a). The distance between adjacent elements is 22.2 mm, about 0.43λ, where λ is the free-space wavelength at 5.8 GHz. Similar to the linear arrays, in the planar array, the 16 × 16 active elements are surrounded by 4 rows of dummy elements to make sure the active elements at different positions with similar active patterns and reflection coefficients.

The measured active reflection coefficients of a central element (no. (8, 8)) corresponding to different scanning angles are shown in Fig. 13(b). Similar to the linear arrays, when the scanning angle increases, the minimums of the active reflection coefficient curves move a little toward low frequency. The active reflection coefficients in the band of 5.73 GHz–5.86 GHz are below −10 dB in all the scanning status.

**Figure 14:**
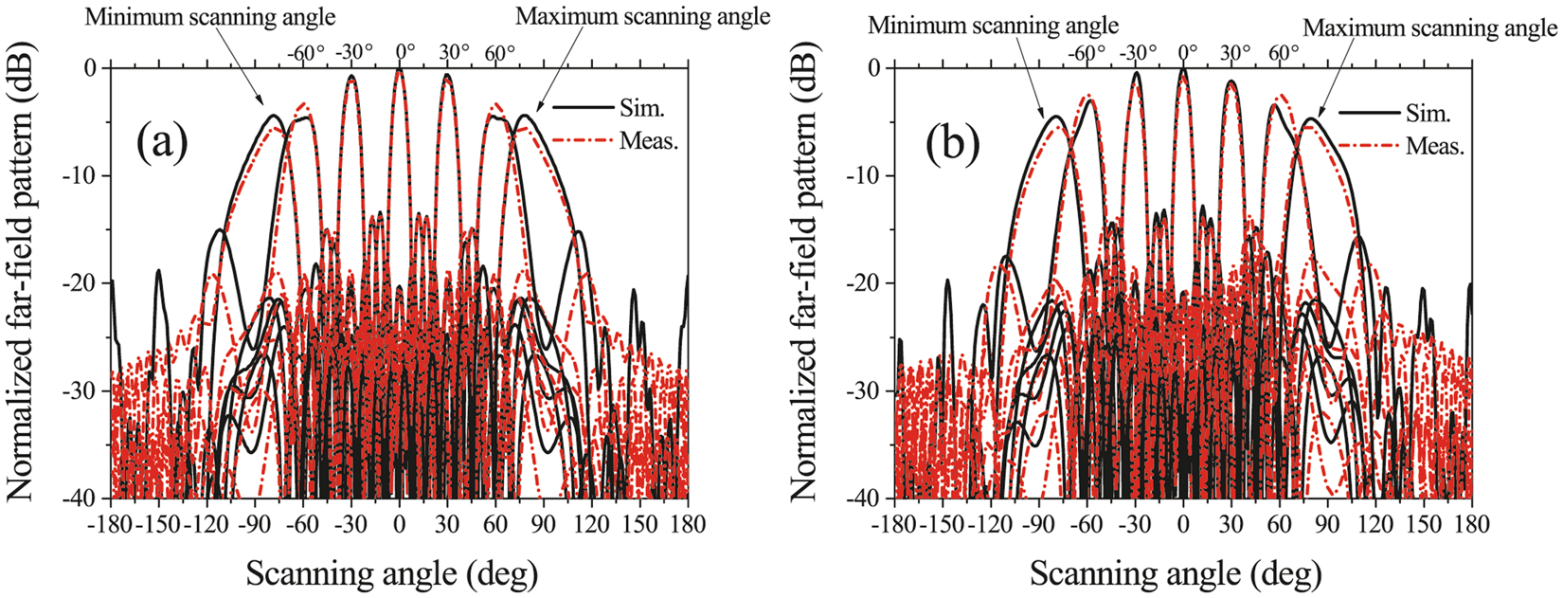
Simulated and measured scanning patterns of the planar array in (**a**) xz plane and (**b**) yz plane at 5.8 GHz. Main beams can scan from less than −77° to more than +77° with a gain fluctuation less than 5 dB. The two scanning planes have similar performances.

The simulated and measured scanning patterns of the planar array are shown in Fig. 14. From Fig. 14, in two special planes, *xz* plane and *yz* plane, the simulated and measured main beams of the planar array can scan from less than −77° to more than +77° with a gain fluctuation less than 5 dB and a SLL less than −10 dB. The two scanning planes have similar performances.

**Figure 15:**
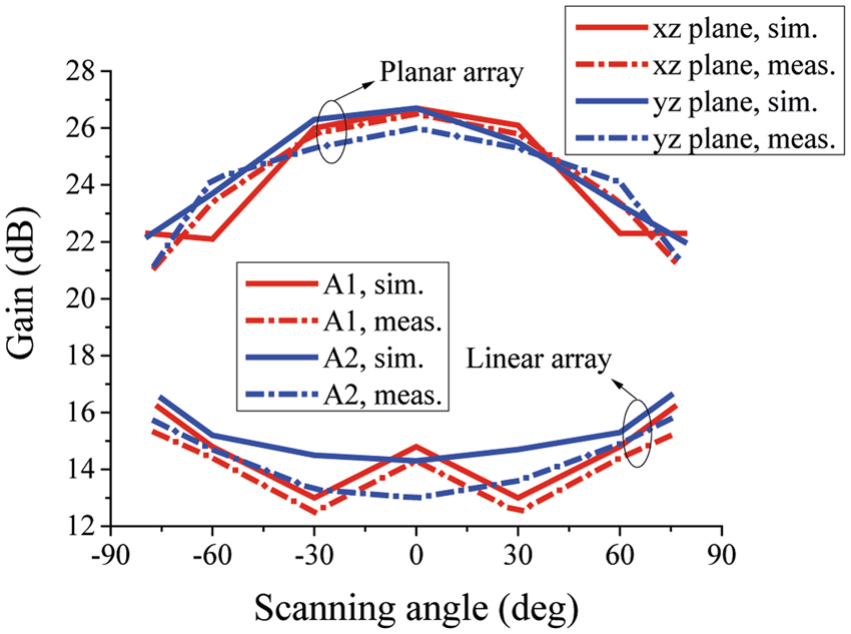
Simulated and measured scanning gains of the proposed linear and planar arrays at 5.8 GHz. Scanning patterns’ maximums of the linear arrays locate closed to the end-fire directions instead of the broadside direction, this characteristic can compensate the attenuation factor cos(θ) of planar array, which makes the directivity of planar array decays not too much when beam scans to end-fire directions.

The simulated and measured scanning gains of the proposed linear and planar arrays are shown in Fig. 15. Because the directivity of planar array has a factor of cos(*θ*), the magnitude of directivity will be 0 when scanning to end-fire directions, which is a challenge to planar wide-angle scanning arrays. From Fig. 15, we can see that the scanning patterns’ maximums of the linear arrays locate closed to the end-fire directions instead of the broadside direction, this characteristic can compensate the attenuation factor cos(*θ*) of planar array, which makes the directivity of planar array decays not too much when beam scans to end-fire directions. Therefore, the planar array can scan its beam to more than 77° with a gain fluctuation within 5 dB.

**Figure 16:**
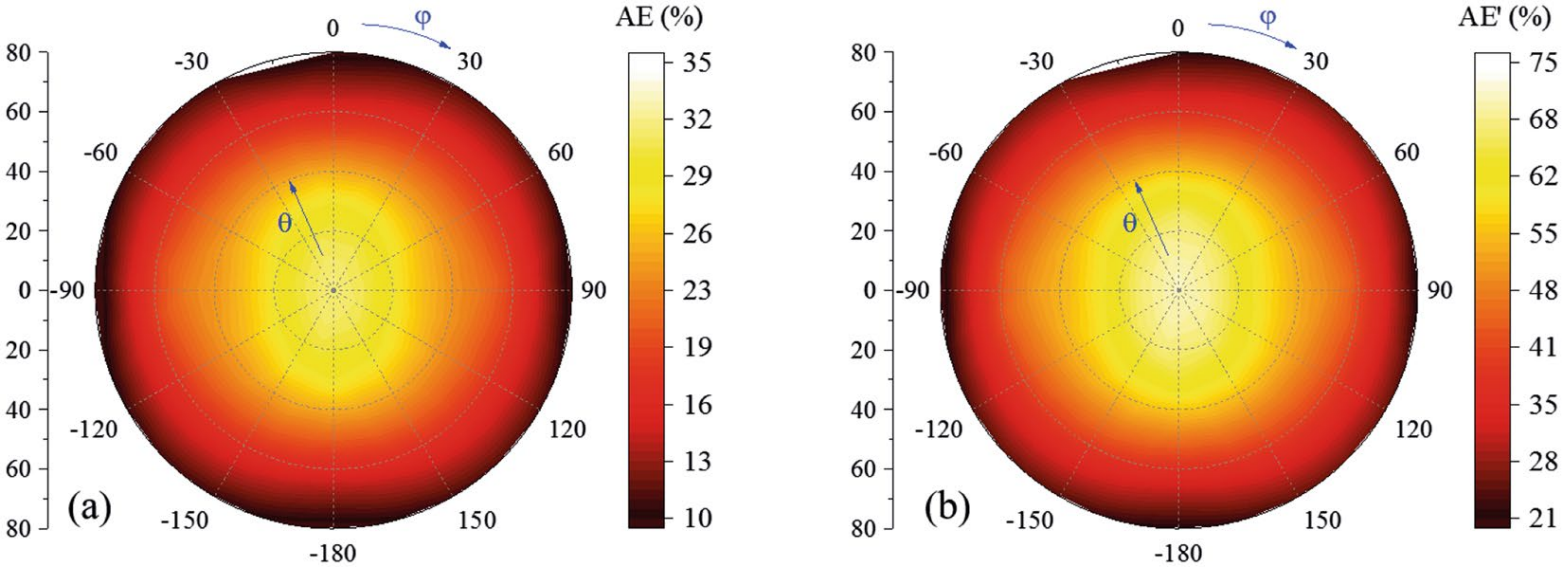
Aperture efficiency of the proposed planar array with 16 × 16 active elements. Aperture efficiencies calculated with and without dummy elements at different scanning angles are shown in (**a**,**b**), respectively.

The aperture efficiency of the proposed planar array with 16 × 16 active elements and four rounds of dummy elements is shown in Fig. 16. The aperture efficiency *η*_*a*_ is calculated with measured scanning gains and array dimension according to

2 $${\eta }_{a}({\theta }_{0},{\phi }_{0})=\frac{G({\theta }_{0},{\phi }_{0}){\lambda }^{2}}{4\pi {A}_{phy}}$$

where *G*(*θ*_0_, *φ*_0_) is the gain when beam is steered to (*θ*_0_, *φ*_0_) direction and *A*_*phy*_ is the physical aperture of calculated array. The aperture efficiency of the proposed planar array with active elements and dummy elements is between 10~35% in an ultra-wide scanning range from approximately −80°~80°. At the near-end-fire directions around 80°, the aperture efficiency is approximately 0.29 times of that at 0°, and this attenuation includes array factor attenuation and impedance mismatching attenuation. For traditional planar arrays, array factor will decay to 0.17 times of that at 0° when scanning to 80° according to cos(θ) attenuation and impedance will change a lot when arrays scan close to end-fire directions, which is the difficulty of wide-angle scanning of planar arrays. Based on the proposed methods, impedance matching at near-end-fire directions is achieved using decoupling cavity and array factor attenuation is partially compensated by element patterns, therefore, aperture efficiency attenuation of the proposed array is smaller than traditional planar arrays, especially at near-end-fire directions. As to the aperture efficiency value, 10~35%, it is not high because of the area of dummy elements. However, the aperture efficiency will increase with array element number. For example, if the array has 64 × 64 active elements, four rounds of dummy elements are still enough to create a similar environment for the active elements at different array positions. In this case, the aperture efficiency will be much higher than the proposed array with 16 × 16 active elements. Because the aim of this paper is to give this novel method and illustrate the method using a practical design example, there is no need to fabricate a very large array. In addition, the aperture efficiency of the proposed array calculated by the area only with active elements is shown in Fig. 16(b), which is approximately two times of Fig. 16(a).

From the above analysis, we can see that both linear and planar TMC arrays can achieve a wide-angle scanning performance. In other word, an excellent wide-angle scanning performance can be obtained by the proposed method. In addition, the wide-angle scanning arrays with TMC elements have a single-layer substrate and a low profile, which is suitable for carrier-based applications.

## Discussion

This study focuses on increasing the field-of-view scanning range of planar arrays. The main idea is to broaden the beam-width of elements, which is the key limiting scanning range. The proposed compact TMC antenna with large ground has a quasi-hemispherical pattern and the beam-width increases with the ground size. Generally, wide-angle scanning arrays have a lot of elements as well as large ground, therefore, the proposed method is suitable for the wide-angle scanning applications. In conclusion, an excellent field-of-view wide-angle-scanning performance both in linear and planar arrays can be obtained by the proposed method, which can be used to guide the design of wide-angle scanning arrays.

## Methods

### Setting the simulation parameters

The TMC antennas and arrays are simulated and optimized using CST Microwave Studio. The operating frequency of the TMC antennas and arrays is set as 5.8 GHz, because the frequency range around 5.8 GHz is an opening spectrum for civilian applications and researches in China. The dielectric substrates of the antennas and arrays have a thickness of 1.6 mm and a relative dielectric constant of 2.2. A copper patch with some grounded vias is printed on the top side of the substrate and fed from the backside with a 50-Ω coaxial probe. The back side of the substrate is full of metal ground.

### Measurement method

The scattering matrixes S_mn_ (m, n = 1, 2, 3, …, 16) of the arrays were measured using an E8361A Vector Network Analyzer. When S parameters of one port are measured, other ports were connected to matched loads. The active reflection coefficients were computed from the measured S parameters of the phased array with the method in refs 25 and 28. Patterns were measured in a microwave anechoic chamber with SATIMO measurement system. When patterns of one element were measured, other ports were connected to matched loads. The scanning performances were calculated with the measured active radiation patterns of the array elements based on the method in refs 25 and 29. Each element of the arrays was fed by a signal of equal amplitude and uniform progressive phase shift *α*. Because all factors that affect the arrays’ performances were taken into consideration through the measured active element patterns, the synthesized array patterns should be the measured array patterns^6^. The main beam of the array is scanned by altering the uniform progressive phase shift *α*. When *α* = ±*k*_*0*_ *d* sin(π/2) = ±*k*_*0*_ *d*, the maximum and minimum scanning angles can be obtained, where *d* is the distance between adjacent elements.

## Electronic supplementary material

Movie S1
