# Supplementary figures and images for: Wide-angle scanning planar array with quasi-hemispherical-pattern elements
Source: Sci Rep. 2017 Jun 2;7:2729. doi: 10.1038/s41598-017-03005-3 (PMC5457415; doi:10.1038/s41598-017-03005-3)

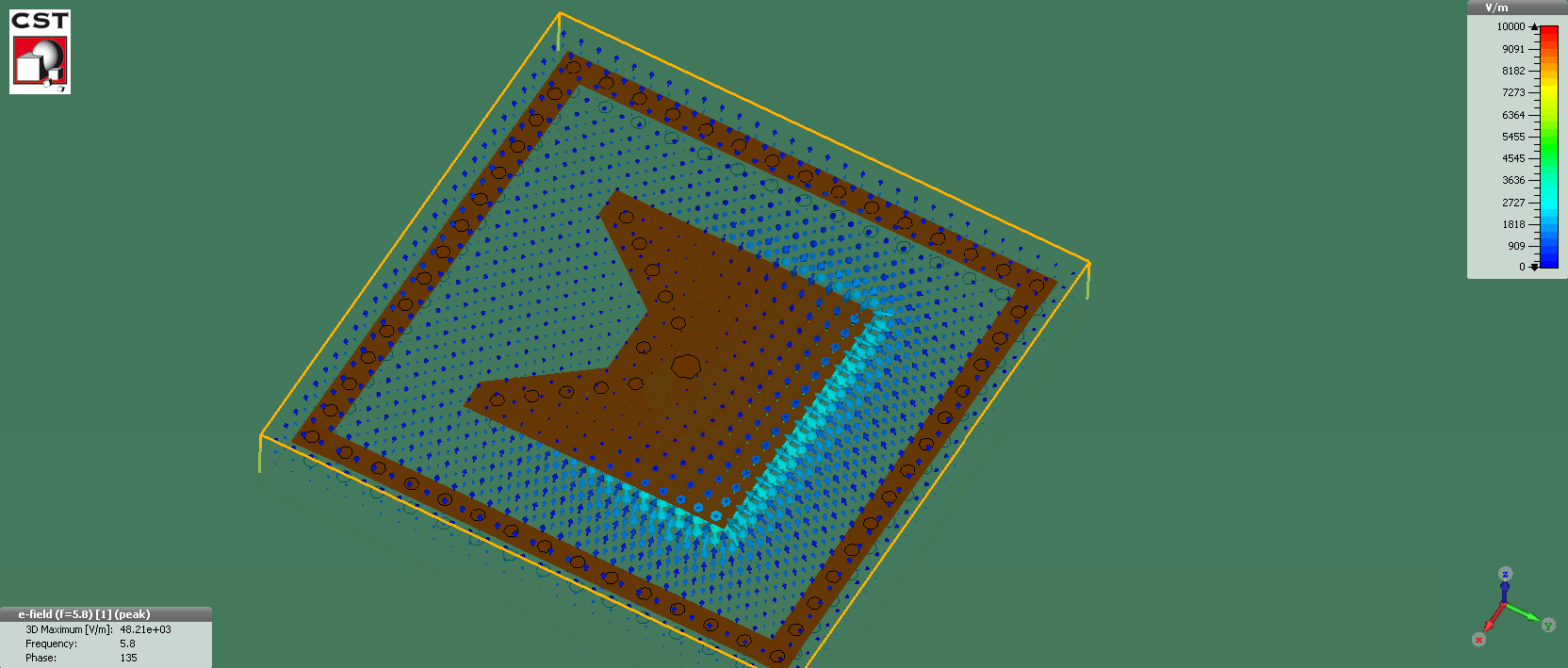

Supplement: Supplementary file 1 — Movie S1 [file 41598_2017_3005_MOESM1_ESM.gif]
